# Mediating Effects of Psychological Independence and Social Support on the Association Between Family Strength and Depression in Young Korean Adults: Cross-Sectional Study

**DOI:** 10.2196/71485

**Published:** 2025-06-27

**Authors:** Sunyoung Kim, Suin Park, Hyunlye Kim, Dabok Noh

**Affiliations:** 1 College of Nursing, Korea University, Seoul, Republic of Korea; 2 Gyeongju Mental Health Welfare Center, Gyeongju, Republic of Korea; 3 Department of Nursing, College of Medicine, Chosun University, Gwangju, Republic of Korea; 4 College of Nursing, Eulji University, 553 Sanseong-daero, Sujeong-gu, Seongnam-si, Gyeonggi-do, 13135, Republic of Korea, 82 317407415

**Keywords:** depression, family strength, psychological independence, social support, young adults

## Abstract

**Background:**

Although family strength is potentially associated with a reduced risk of depression, little is known about the underlying pathways and mediating factors.

**Objective:**

This study aimed to investigate the mediating effects of psychological independence and social support on the relationship between family strength and depression in young adults.

**Methods:**

A cross-sectional web-based survey was conducted among 1,000 young Korean adults aged 19 to 24 years. We used a web-based survey agency to recruit participants using an independent panel and quota sampling, with stratification based on gender and age. The participants completed self-reported questionnaires that assessed family strength, psychological independence, social support, and depression. To examine the mediating effects of psychological independence and social support on the relationship between family strength and depression, we performed path analysis with AMOS 26 software (IBM Corp) using maximum standard likelihood estimation.

**Results:**

The path analysis revealed that gender (female) had a direct positive effect on depression (*β*=.09, *P*=.004) and an indirect negative effect on depression through social support (β=−.03, *P*=.001). Although there were no significant direct effects of living status (with parents) on depression, it had a significant and positive indirect effect through psychological independence (*β*=.03, *P*=.001). Family strength had a significant and negative direct effect on depression (β=−0.19, *P*=.001) and significant indirect and negative effects through psychological independence and social support (β=−0.17, *P*=.001). Therefore, the overall effect of family strength on depression was significantly negative (β=−0.37, *P*=.001). Psychological independence influenced depression both directly (β=−0.16, *P*=.001) and indirectly through social support (β=−0.12, *P*=.001), and social support influenced depression directly (β=−0.21, *P*=.001). The overall model explained 23% of the total variance in depression.

**Conclusions:**

The findings highlight that gender, living with parents, family strength, psychological independence, and social support in reduce depression among young adults. Additionally, the mediating effects of psychological independence and social support on the relationship between family strength and depression were significant in this population. Therefore, strategies to increase psychological independence and social support could reduce the risk of depression in young adults who have low family strength.

## Introduction

The World Mental Health Report claims that of all mental disorders, depression has the strongest burden [1]. A national US survey found that three-quarters of all lifetime cases of depression started before people were 24 years old [2]. In South Korea, depression in young adults has become increasingly prevalent in recent years. In March 2022, Korea’s National Mental Health Survey found a depression rate of 18.5% in people in their 20s [3]. Early adulthood or “emerging adulthood,” which was recently recognized as a developmental psychology life stage, is a crucial early stage for preventive interventions to treat depression [4].

Family strength promotes positive growth; therefore, children raised in strong families are more likely to be healthy and develop strong family units as adults [5]. Sittner et al [6] described family strength as “commitment, appreciation and affection, positive communication, enjoyable time together, a sense of spiritual well-being, and the ability to cope with stress and crisis.” Other studies have also found that family strength can reduce the likelihood of depression in family members [7-9].

Young adulthood is defined as being between the ages of 19 and 24 years [10]. This is generally a period of transition from childhood dependence and adolescence to assuming adult roles and responsibilities [11]. While receiving continued support from their parents, young adults are also seeking to establish their identities and prepare for independence [12]. Psychological independence in young adults is defined as autonomous thinking and behavior and concurrently maintaining a positive, secure relationship with their parents based on attachment [13]. Higher psychological independence is associated with lower rates of depression in young adults [14].

Social support can be an important protective factor against mental health problems in emerging adulthood, which is a transitional period in which people go through substantial changes in their social roles and responsibilities [4]. Previous studies have revealed that higher perceived social support is associated with lower levels of depression in young adults [15,16].

Psychological independence could also play a mediating role between family strength and depression. Previous studies have found that higher perceived family strength is associated with greater psychological independence [17], and dysfunctional family lives and conflicted dependence on parents are associated with higher levels of suicidal ideation [18]. Psychological independence was found to mediate the relationship between family strength and depression [19].

Social support may also mediate the relationship between family strength and depression. For example, a previous study found that family strength mediated through social support had a significant effect on happiness in young adults [20], and another study found that social support mediated the relationship between family strength and psychological well-being in children [21].

The theoretical framework of this study is based on the Social Support Theory Model, which emphasizes the role of social support in enhancing an individual’s ability to cope with stress and positively influencing their health [22]. A previous study found that individuals with high levels of psychological independence are more actively engaged in social relationships and are more likely to receive support from others, suggesting that psychological independence is a significant factor in promoting social support [23]. Another study emphasized that social support increases psychological stability and reduces the perception of stress, thereby lowering the risk of depression [24]. Psychological independence facilitates social support, which in turn reduces the risk of depression, ultimately supporting the mental health of young adults through this mechanism.

**Figure 1.**
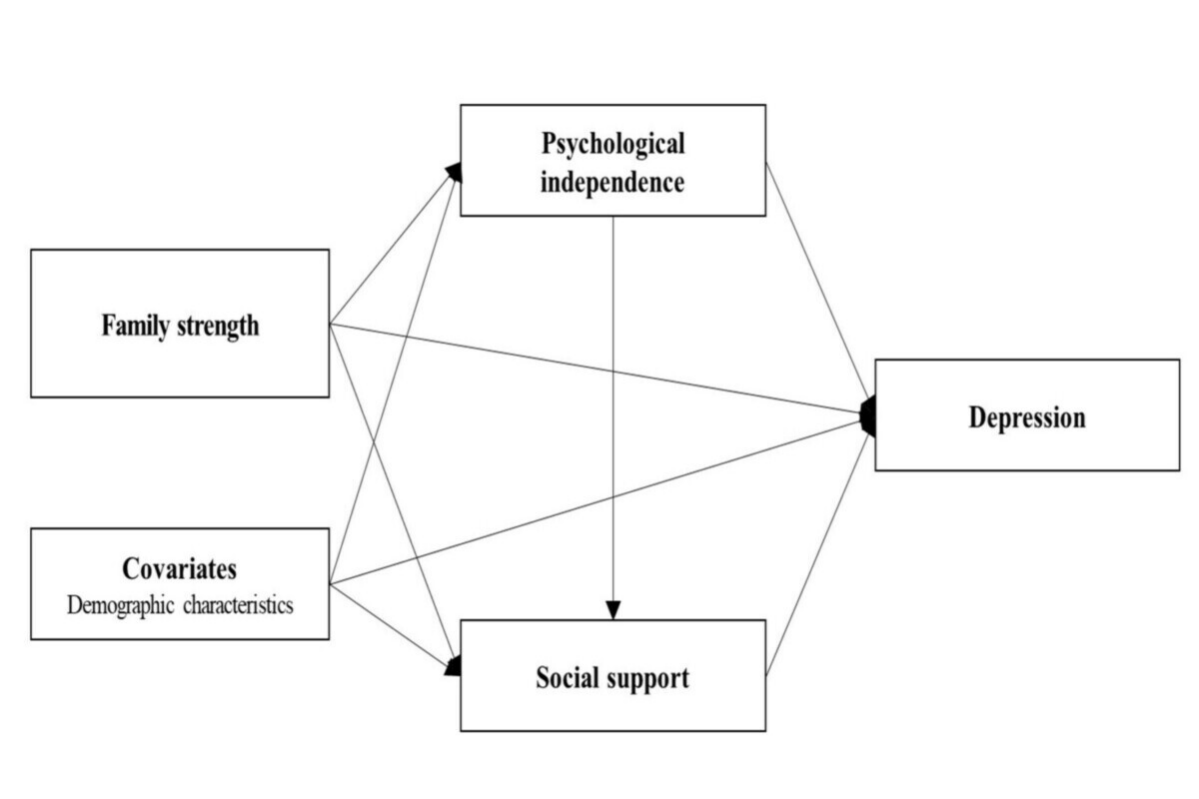
Hypothetical study model: mediating effects of psychological independence and social support on the relationship between family strength and depression among young Koreans adults aged 19‐24 years. Arrows indicate the hypothesized causal relationships between variables.

These study results suggest that psychological independence and social support could mediate the relationship between family strength and depression; however, little is known regarding these structural relationships. In this study we conducted a path analysis of the relationships among family strength, psychological independence, social support, and depression in young adults. Figure 1 shows our hypothesized model, in which family strength influences depression, with this relationship being mediated by psychological independence and social support.

## Methods

### Study Design and Participants

A cross-sectional web-based survey was conducted among 1,000 young Korean adults aged 19‐24 years. This study adhered to the STROBE (Strengthening the Reporting of Observational Studies) guidelines, ensuring rigorous compliance with the reporting standards for cross-sectional studies. Using the G-power version 3.1.9.4 program [25], the required sample size was calculated based on a significance level of 5%, statistical power of 85%, an effect size of 0.02 (small), and 11 predictors. The minimum required sample size was determined to be 945. Considering a 6% dropout rate, the final sample size was set at 1,000.

### Measures

#### Family Strengths

We used the Korea Family Strengths Scale II [26] to assess family strength within the Korean cultural context. This scale comprises 5 dimensions: family resilience; valuing each other and acceptance; qualitative bonding; economic stability and cooperation; and caring about the community, within which 22 self-reported items are scored on a 5-point Likert scale ranging from 1 (strongly disagree) to 5 (strongly agree). An example questionnaire item is “My family trusts each other.” Scores range from 22 to 110, with higher scores indicating greater family strength. Cronbach α coefficient was 0.94 in a prior study [26] and 0.97 in this study.

#### Psychological Independence

Psychological independence was evaluated using the Psychological Independence Scale [12], which comprises 3 factors: supportive relationship with parents; voluntary decision-making; and self-reliability. The “supportive relationship with parents” factor has 6 items related to feeling a sense of being accepted as they are, having their decisions respected, and having their opinions valued when solving family issues [12]. The “voluntary decision-making” factor has 6 items related to autonomously acting based on one’s own thoughts or approaches rather than parental opinions in decision-making [12]. The “self-reliance” factor has 6 items related to having confidence in one’s career path, life goals, and potential, and using this confidence to establish personal goals and plans [12]. The scale has 18 self-report items that are scored on a 5-point Likert scale ranging from 1 (strongly disagree) to 5 (strongly agree), with 6 negatively worded items being reverse-coded. An example questionnaire item is “I have my own life goals.” Scores range from 18 to 90, with higher scores indicating greater psychological independence. Cronbach α coefficient was 0.83 in a prior study [12] and 0.83 in this study.

#### Social Support

The Korean version of the Social Provision Scale [27], which was translated into Korean by Yoo and Lee [28], was used to assess perceived social support. The scale has 6 factors—guidance, reassurance of worth, social integration, attachment, opportunity for nurturance, and reliable alliance—to assess relationships with friends, family, coworkers, community members, and others. The 24 items are rated on a 4-point Likert scale ranging from 1 (strongly disagree) to 4 (strongly agree). An example questionnaire item is “There are people I can depend on to help me if I really need it.” Of these 24 items, the 12 negatively worded items are reverse-coded. Scores range from 24 to 96, with higher scores indicating greater perceived social support. Cronbach α coefficient was 0.92 in a prior study [27] and 0.93 in this study.

#### Depression

Depression was assessed using the Korean version of the Center for Epidemiologic Studies Depression Scale-Revised (K-CESD-R) [29], which is the Korean translation of CESD-R [30]. The K-CESD-R has 20 self-report items scored on a 5-point Likert scale ranging from 0 (less than 1 day in the last week) to 4 (nearly every day in the last 2 weeks). An example questionnaire item is “I could not shake off the blues.” Scores range from 0 to 80, with higher scores indicating a higher level of depression. The Cronbach α coefficient for the K-CESD-R was 0.98 in a prior study [29] and 0.95 in this study.

#### General Characteristics

We also collected participant characteristics, including gender, age, living status, educational level, religion, job, household economic status, and residential area.

### Data Collection

A web-based survey agency distributed research descriptions and survey links via email to an independent panel of young Korean adults aged 19‐24 years. They collected data using quota sampling with gender and age stratification, ensuring an even distribution of participants to minimize potential sampling bias. Participants reviewed the survey information, provided informed consent, and accessed the web-based survey via the provided URL. The data were collected from the 1000 participants between October 24, 2022, and October 31, 2022.

### Data Analyses

The data were analyzed using SPSS (version 26.0) and AMOS (version 26.0) software (IBM Corp). Descriptive analyses were generated for demographic characteristics, family strength, psychological independence, social support, and depression. Independent 2-tailed *t* tests, ANOVA with the Scheffe post hoc test, and Pearson correlations were used to analyze family strength, psychological independence, social support, and depression based on demographic characteristics. To examine the mediating effects of psychological independence and social support on the relationship between family strength and depression, we performed path analysis with AMOS 26 software using maximum standard likelihood estimation.

### Ethical Considerations

The study protocol was reviewed by the institutional review board of Eulji University (approval number: EU22-71, date of approval: October 17, 2022). Participants were provided with study information on the first screen of the web-based questionnaire. After reading the information, they provided electronic consent by clicking the consent button. To prevent duplicate submissions, a system was implemented to detect and block multiple responses; if a repeat attempt was made, a notification was displayed and access was restricted. If a participant withdrew from the survey—for example, by closing the website—their data were automatically discarded. Upon survey completion, all data were processed automatically to ensure anonymity and were coded using nonidentifiable markers. Nonmonetary incentives emphasized the importance of participants’ contributions in advancing mental health research and intervention development.

## Results

### Main Variables Based on Demographic Characteristics

Table 1 shows the data for demographic characteristics, family strength, psychological independence, social support, and depression. Out of 1000 participants in the study, 520 (52%) were male, the mean age was 21.68 (SD 1.68) years, and more than two-thirds lived with their parents (n=676, 67.6%). Most of the 1,000 participants had a college education or higher (n=844, 84.4%), had no religion (n=720, 72.0%), and had no job (n=826, 82.6%). The self-reported household economic status of 1,000 participants was middle 434 (43.4%), low 284 (28.4%), and high 282 (28.2%), with nearly half living in big cities 489 (48.9%), followed by small- or medium-sized cities 404 (40.4%), and rural areas 107 (10.7%).

Compared to females, the males reported higher family strength (*t*_998_=5.03, *P*<.001) and lower social support (*t*_997.45_=−2.16, *P*=.031) and depression (*t*_998_=−3.69, *P*<.001). Participants not living with their parents showed higher psychological independence than those living with their parents (*t*_998_=2.69, *P*=.007). Compared to high school graduates, participants with a college education or higher had higher levels of family strength (*t*_998_=−4.80, *P*<.001), psychological independence (*t*_998_=−2.72, *P*=.007), and social support (*t*_998_=−2.86, *P*=.004), and lower levels of depression (*t*_190.21_=3.46, *P*=.001). Religious participants had higher family strength than those who were nonreligious (*t*_998_=−3.77, *P*<.001). Participants with low self-reported household economic status had significantly lower family strength than those with middle or high economic status (*F*_2, 997_ =77.55, *P*<.001). Participants with low self-reported household economic status had the lowest levels of psychological independence (*F*_2, 997_=23.84, *P*<.001) and social support (*F*_2, 997_ =22.23, *P*<.001), and the highest level of depression (*F*_2, 997_ =16.51, *P*<.001), followed by those with middle or high household economic status. Participants living in big cities had higher family strength than those living in small- or medium-sized cities and rural areas (*F*_2, 997_ =3.42, *P*=.033). Participants living in rural areas had lower levels of psychological independence than those living in big cities and small- or medium-sized cities (*F*_2, 997_=4.69, *P*=.009).

**Table 1.** Family strength, psychological independence, social support, and depression according to demographic characteristics (N=1000).

Variables		Values	Family strengths			Psychological independence			Social support			Depression		
			Mean (SD)	Statistics	*P* value	Mean (SD)	Statistics	*P* value	Mean (SD)	Statistics	*P* value	Mean (SD)	Statistics	*P* value
**Gender**				*t*_998_=5.03	<.001		*t*_998_=1.66	.10		*t*_997.45_=–2.16	.03		*t*_998_=–3.69	<.001
	Male	n=520 (52.0%)	81.46 (1 7.36)			65.08 (9.49)			71.46 (12.38)			12.55 (15.43)		
	Female	n=480 (48.0%)	76.00 (16.98)			64.05 (10.34)			73.07 (11.16)			16.10 (14.98)		
**Age (years)**		Mean 21.68 (SD 1.68)	—^a^	*r*_998_=–0.05	.11	—	*r*_998_=–0.03	.40	—	*r*_998_=–0.04	.23	—	*r*_998_=–0.06	.07
**Living status**				*t*_998_=–0.30	.76		*t*_998_=2.69	.01		*t*_998_=1.46	.15		*t*_998_=1.32	.19
	Without parents	n=324 (32.4%)	78.60 (17.69)			65.80 (9.48)			73.02 (11.40)			15.18 (15.35)		
	With parents	n=676 (67.6%)	78.95 (17.25)			64.00 (10.07)			71.86 (12.02)			13.81 (15.29)		
**Education level**				*t*_998_=–4.80	<.001		*t*_998_=–2.72	.01		*t*_998_=–2.86	.004		*t*_190.21_=3.46	.001
	High school	n=156 (15.6%)	72.78 (18.71)			62.61 (9.61)			69.76 (12.92)			18.88 (18.79)		
	College or higher	n=844 (84.4%)	79.96 (16.90)			64.95 (9.93)			72.69 (11.57)			13.40 (14.43)		
**Religious views**				*t*_998_=–3.77	<.001		*t*_998_=–1.74	.08		*t*_998_=–1.67	.10		*t*_998_=–0.03	.97
	No	n=720 (72.0%)	77.56 (17.56)			64.25 (10.04)			71.84 (11.96)			14.25 (15.02)		
	Yes	n=280 (28.0%)	82.14 (16.49)			65.46 (9.55)			73.24 (11.44)			14.28 (16.06)		
**Having a job**				*t*_998_=1.00	.32		*t*_998_=0.87	.39		*t*_998_=0.03	.97		*t*_998_=0.96	.34
	No	n=826 (82.6%)	79.09 (17.42)			64.71 (10.00)			72.24 (12.03)			14.47 (15.30)		
	Yes	n=174 (17.4%)	77.64 (17.20)			63.99 (9.49)			72.21 (10.86)			13.25 (15.36)		
**Household economic status**				*F*_2,997_=77.55	<.001, a<b,c^b^		*F*_2,997_=23.84	<.001, a<b<c		*F*_2,997_=22.23	<.001, a<b<c		*F*_2,997_=16.51	<.001, c<b<a
	Low	n=284 (28.4%)	68.89 (17.11)			61.46 (10.55)			68.65 (12.81)			19.08 (17.77)		
	Middle	n=434 (43.4%)	81.68 (15.60)			65.08 (9.40)			72.76 (11.03)			12.46 (13.45)		
	High	n=282 (28.2%)	84.49 (1 6.12)			66.97 (9.22)			75.03 (11.12)			12.16 (14.29)		
**Residential area**				*F*_2,997_=3.42	.03, e,*f*<d^c^		*F*_2,997_=4.69	.01, *f*<d,e		*F*_2,997_=3.00	.05		*F*_2,997_=1.15	.32
	Big city	n=489 (48.9%)	80.19 (17.11)			65.09 (9.80)			72.75 (11.86)			13.52 (14.14)		
	Small- or medium-sized city	n=404 (40.4%)	77.95 (17.18)			64.69 (9.99)			72.29 (11.78)			14.85 (16.34)		
	Rural area	n=107 (10.7%)	76.03 (18.96)			61.88 (9.78)			69.66 (11.67)			15.38 (16.41)		

a Not applicable.

b a: Low; b: Middle; c: High

c d:Big city; e: Small- or medium- sized city; f: Rural area

### Levels of Main Variables

Table 2 shows the results for family strength, psychological independence, social support, and depression. The respective mean family strength, psychological independence, social support, and depression scores were 78.84 (SD 17.38), 64.59 (SD 9.91), 72.23 (SD 11.83), and 14.26 (SD 15.31).

**Table 2.** Family strength, psychological independence, social support, and depression (N=1000).

Characteristics	Mean (SD)	Observed range	Possible range
Family strengths	78.84 (17.38)	22‐110	22‐110
Psychological independence	64.59 (9.91)	30‐90	18‐90
Social support	72.23 (11.83)	26‐96	24‐96
Depression	14.26 (15.31)	0‐80	0‐80

### Path Analysis on the Determinants of Depression

**Figure 2.**
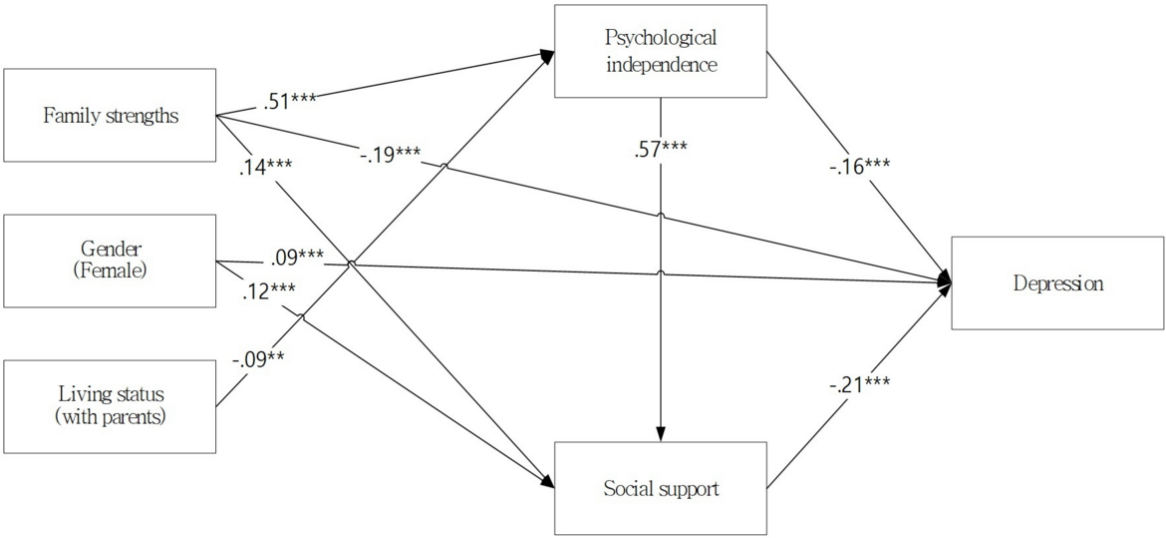
Final path diagram for depression in young adults: The direct and indirect effects of family strengths, social support, psychological independence, and demographic factors (gender and living status) on depression. Path coefficients are presented as standardized beta values with significant levels (*P<.05, **P<.01, *** *P*<.001).

Our previous bivariate analyses found that gender, living status, educational level, household economic status, and residential area were significantly associated with psychological independence, social support (mediating variables), and depression (dependent variable). Therefore, these demographic variables—gender, living status, educational level, household economic status, and residential area—were included as covariates in the initial model of this study. However, of the demographic variables, household economic status was excluded in the final model because it was closely related to educational level, and to better enhance the model fit, the residential area was excluded due to the lack of meaningful results. Figure 2 shows the final path diagram for the modified model.

The final model had a good fit to the data (minimum discrepancy function/degrees of freedom=3.000, goodness of fit index=0.998, comparative fit index=0.998, Tucker Lewis index=0.990, standardized root-mean-squared residual=0.015, and root-mean-square error of approximation=0.028).

The direct, indirect, and total effects of the final model are shown in Table 3. Gender (female) had a direct positive effect on depression (*β*=.09, *P*=.004), and an indirect negative effect on depression through social support (β=−0.03, *P*=.001). Although there were no significant direct effects of living status (with parents) on depression, it had a significant and positive indirect effect through psychological independence (*β*=.03, *P*=.001).

Family strength had a significant and negative direct effect on depression (β=−0.19, *P*=.001), and significant indirect negative effects through psychological independence and social support (β=−0.17, *P*=.001). Therefore, the overall effect of family strength on depression was significantly negative (β=−0.37, *P*=.001).

Our analysis revealed that there were significant psychological independence and social support mediation effects. Psychological independence influenced depression both directly (β=−0.16, *P*=.001) and indirectly through social support (β=−0.12, *P*=.001), and social support influenced depression directly (β=−0.21, *P*=.001). The overall model explained 23% of the total variance in depression.

**Table 3.** Direct, indirect, and total effects of the final modified model.

Path	Direct	Indirect	Total
	β (*P* value)	β (*P* value)	β (*P* value)
Living status (with parents) → psychological independence (SMC^a^=.263)	−0.09 (.001)	—^b^	−0.09 (.001)
Family strength → psychological independence (SMC=.263)	0.51 (.001)	—	0.51 (.001)
Gender (female) → social support (SMC=.430)	0.12 (.001)	—	0.12 (.001)
Living status (with parents) → social support (SMC=.430)	—	−0.05 (.001)	−0.05 (.001)
Family strength → social support (SMC=.430)	0.14 (.001)	0.29 (.001)	0.43 (.001)
Psychological independence → social support (SMC=.430)	0.57 (.001)	—	0.57 (.001)
Gender (female) → depression (SMC=.230)	0.09 (.004)	–0.03 (.001)	0.07 (.04)
Living status (with parents) → depression (SMC=.230)	—	0.03 (.001)	0.03 (.001)
Family strength → depression (SMC=.230)	−0.19 (.001)	−0.17 (.001)	−0.37 (.001)
Psychological independence → depression (SMC=.230)	−0.16 (.001)	−0.12 (.001)	−0.28 (.001)
Social support → depression (SMC=.230)	−0.21 (.001)	—	−0.21 (.001)

a SMC: squared multiple correlations for structure equations.

b Not applicable.

## Discussion

### Principal Findings

This study examined the mediating effects of psychological independence and social support on the relationship between family strengths and depression among young adults. The findings indicate that both factors serve as significant mediators.

The mean depression score (14.26, SD 15.31) in our young adult study sample was slightly lower than that of other studies using the K-CESD-R. Yi et al [31] reported a mean depression score of 15.55 (SD 11.87) in Korean female college students, and Kim and Park [32] reported a mean score of 17.31 (SD 8.29) in Korean college students. As a cutoff score of 13 indicates a risk of clinical depression [29], most studies have found the average depression level in Korean young adults to be relatively high. This could be a result of job insecurity [33], economic instability, or the lack of quality, stable jobs, and employment opportunities [34] for young Korean adults. In addition, the relatively large SD of 15.31 observed in this study highlights significant variability in depression levels among participants, implying the existence of a subgroup with particularly high levels of depression. These findings emphasize the need for more detailed analyses of depression levels in future research to better understand and address this issue.

Our results confirm previous findings regarding significant gender differences in depression levels. Kim et al [35] and Salk et al [36] reported higher levels of depression in females in globally representative samples. Our study found that these gender differences are more pronounced in Korean society, likely due to the unique cultural norms present in Confucian-oriented societies. In Korea, Confucian traditions emphasize harmony, social order, and hierarchy, which restrict individual emotional expression and particularly lead men to suppress their emotions to a greater degree than women [37], thereby contributing to a lower reported depression level among men. In Confucian cultural contexts, young adult women experience elevated levels of depression, primarily attributable to the dual burden of fulfilling traditional familial roles and conforming to heightened societal expectations [38]. This dual burden likely explains the higher depression levels observed in the female participants of our study.

Our results also found that the perceived social support score for females was significantly higher than that of males. A qualitative analysis reported that the types of preferred social support also differed by gender in young adults [39]. They found that males tended to prioritize social network support, which helped them forget and control their emotional distress by engaging in activities, such as having fun, whereas females prioritized social support, which helped them talk about their problems and analyze their emotional distress. The social support measurement scale we used assessed guidance, reassurance of worth, social integration, attachment, opportunity for nurturance, and reliable alliance in relationships with friends, family, coworkers, community members, and others [28]. Therefore, as the measurement tool’s characteristics may have aligned more closely with women’s preferred social support types, there may have been higher perceived social support levels for women than for men. However, further research may be needed to determine the gender differences in social support.

Our research results also revealed that young adults living with their parents had significantly lower levels of psychological independence than those not living with their parents. Our findings align with those of prior research conducted with Japanese university students, suggesting that the negative impact of cohabiting with parents on psychological independence may be a shared phenomenon within the Confucian-influenced East Asian cultural sphere [40]. This may be because of the current socioeconomic conditions in Korea, as factors such as increased job insecurity and rising housing costs are preventing many young adults from becoming independent from their parents [41]. Older generations in Korea also tend to have family values that consider it natural to continue looking after their children [41]. Consequently, many young adults who continue to live with their parents are also dependent on their parents, which could impact their psychological independence.

Our results revealed a direct negative effect of family strength on depression in young adults. Family strength comprises 5 factors: family resilience, valuing each other and acceptance, qualitative bonding, economic stability and cooperation, and caring about the community [26]. Consistent with our findings, a study targeting college students reported a negative direct effect of family strength on depression [19]. Other studies targeting adolescents have also reported that higher perceived family resilience was associated with lower depression [42,43]. Rahman et al [44] also reported that higher family cohesion, that is, the bond and interconnectedness between family members, resulted in lower depressive symptoms in young adults. Because the results suggested that young adults with weak family strength are possibly more vulnerable to depression, psychiatric and mental health nurses should assess family strength when dealing with depression and develop preventive interventions for young people who have poor family strength.

The path analysis revealed that family strength influenced psychological independence, which, in turn, influenced depression. This result was also similar to previous studies on college students [19], and a previous study on Korean high school students that found that the lower the perception of family strength, the lower the psychological independence [17]. An earlier study on Korean college students [14] also reported that the lower the psychological independence level, the higher the level of depression. Jeon [45] examined the relationship mechanism between psychological independence and depression, finding that the lower the psychological independence from the parents, the higher the attachment anxiety (fear of rejection or abandonment) and the attachment avoidance (persistent avoidance or discomfort with intimacy), and the higher the depression levels. Therefore, to prevent depression, the psychological independence of young people from families that have low family strength should be assessed and relevant interventions provided.

“Psychological independence” is defined as a state in which young adults exhibit autonomy in their thoughts and behaviors while maintaining supportive relationships with their parents based on stable attachment [12]. However, parenting methods such as helicopter parenting, which is characterized by overprotective and controlling behavior toward children [46], may impair the development of independence and autonomy in young adults [47]. In their study on college students, Kim and Park [48] found that the higher the perceived level of helicopter parenting, the lower the level of assertiveness, and the higher the level of depression. Vigdal and Brønnick’s [46] systematic review also concluded that helicopter parenting is associated with depression in young adults. Therefore, it is possible that rather than overprotective and controlling parenting, providing psychological independence through supportive parental relationships could reduce the possibility of depression in young adults.

This study found that social support played a mediating role between family strength and depression, which again suggests that interventions that seek to improve social support could reduce the prevalence of depression in young adults with weak family strength. Consistent with this finding, Cano et al [49] reported that interpersonal resources, such as family cohesion and social support, are associated with depression in young adults, and Mecha et al [50] found that perceived social support from family, friends, and significant others is associated with depression in young adults. Young adults with weak family strengths, such as those in out-of-home care, often struggle to receive social support from their families. Therefore, interventions are needed that promote social support through relationships with friends and significant others.

### Limitations

This study has several limitations. First, it was limited to Korean young adults aged 19 to 24 years recruited through a web-based panel; therefore, the sample may not have fully represented the young emerging adult population in Korea. Future studies should recruit individuals from various age groups and geographic regions to enhance generalizability. Second, due to the cross-sectional nature of this study, causal inferences could not be made. The directionality of the relationships between family strength, psychological independence, social support, and depression variables, therefore, could not be determined. Because economic and social conditions change over time, longitudinal studies are needed to track changes in depression levels and related factors. Third, the use of self-reported measures could lead to social desirability and recall bias, which could have resulted in overreporting or underreporting. To mitigate this, participants were provided with clear instructions prior to commencing the web-based survey via mobile devices, ensuring they had sufficient time to respond thoughtfully. Finally, the analysis of the factors anticipated to influence depression in this study yielded an explanatory power of 23%. Given the potential existence of additional variables impacting depression among young adults that were not included in this investigation, future research should explore other factors that may contribute to depression to provide a more comprehensive understanding.

### Practical Implications

Our findings suggest that strategies to enhance psychological independence and social support can significantly reduce depression among young adults with weak family strengths, underscoring the need for tailored preventive interventions. Mental health professionals should assess family strengths and depression levels to identify high-risk groups and provide targeted support. Psychological independence can be fostered through coaching-based programs involving influential peers or professionals outside the family [40], while social connections can be strengthened through group activities and mentoring initiatives [51]. Although the government provides professional psychological counseling services, these are not specifically designed for young adults, and limited awareness leads to underutilization. Therefore, it is essential to implement mental health support programs in universities and workplaces and improve accessibility through comprehensive promotion efforts.

### Conclusions

Although the average depression scores in this study sample were slightly lower than those in previous Korean studies, they still indicated a high risk of depression, with young adult females having higher depression levels than young adult males. Young adults living with their parents were found to have lower psychological independence than those not living with their parents. The main finding of this study was the indirect effect of family strength on depression mediated through psychological independence and social support. This result suggests that strategies targeting these mediating factors could reduce the risk of depression in young adults who have weaker family strength. Mental health professionals should assess young adult family strengths, psychological independence, and social support to provide more focused preventive interventions for depression.

## Abbreviations

K-CESD-R: Korean version of the Center for Epidemiologic Studies Depression Scale-Revised
STROBE: Strengthening the Reporting of Observational Studies
